# Mechanical stress activates NMDA receptors in the absence of agonists

**DOI:** 10.1038/srep39610

**Published:** 2017-01-03

**Authors:** Mohammad Mehdi Maneshi, Bruce Maki, Radhakrishnan Gnanasambandam, Sophie Belin, Gabriela K. Popescu, Frederick Sachs, Susan Z. Hua

**Affiliations:** 1 Department of Physiology and Biophysics, University at Buffalo, Buffalo, New York, 14260, USA; 2 Department of Mechanical and Aerospace Engineering, University at Buffalo, Buffalo, New York 14260, USA; 3 Department of Biochemistry, University at Buffalo, Buffalo, New York 14260, USA

## Abstract

While studying the physiological response of primary rat astrocytes to fluid shear stress in a model of traumatic brain injury (TBI), we found that shear stress induced Ca^2+^ entry. The influx was inhibited by MK-801, a specific pore blocker of N-Methyl-D-aspartic acid receptor (NMDAR) channels, and this occurred in the absence of agonists. Other NMDA open channel blockers ketamine and memantine showed a similar effect. The competitive glutamate antagonists AP5 and GluN2B-selective inhibitor ifenprodil reduced NMDA-activated currents, but had no effect on the mechanically induced Ca^2+^ influx. Extracellular Mg^2+^ at 2 mM did not significantly affect the shear induced Ca^2+^ influx, but at 10 mM it produced significant inhibition. Patch clamp experiments showed mechanical activation of NMDAR and inhibition by MK-801. The mechanical sensitivity of NMDARs may play a role in the normal physiology of fluid flow in the glymphatic system and it has obvious relevance to TBI.

N-Methyl-D-aspartic acid receptors (NMDARs) are mediators of synaptic activity in the brain. Inappropriate activation of NMDARs produces neuronal dysfunctions including cell death^1^. During traumatic brain injury (TBI), there is an elevation of Ca^2+^ in glia and neurons and the source of the Ca^2+^ influx has been postulated to include NMDARs, cationic mechanosensitive ion channels (MSCs) or voltage-gated Ca^2+^ channels^2^,^3^. Several studies showed mechanical modulation of NMDARs, but most papers assumed that this occurred by modulation of agonist affinity^4^,^5^. Martinac’s group, however, showed that reconstituted NMDARs can be activated by bilayer tension in the absence of agonists^6^. We tested the mechanical properties of NMDARs *in vitro* in the adult rat astrocyte preparation and could reproduce the results. This mechanical sensitivity of the channels suggests that patch clamp data may need to be reexamined since patched membranes are under high tension due to the binding of membrane to the glass^7^,^8^.

Our primary assay applied fluid shear stress to cultured primary rat astrocytes in a microfluidic chamber where we monitored Ca^2+^ using fluorescence microscopy. We applied precise shear pulses using a high speed pressure-servo^9^. A shear pulse of 23 dyn/cm^2^ for only 10 ms caused a transient rise in Ca^2+^ that slowly grew, peaking in ~4 s (Fig. 1a/control panel, 1b). The late Ca^2+^ peak is evidence of a “memory” of the transient stimulus^10^. However, there was no significant latency between the beginning of the stimulus and the beginning of the response. We presumed that the Ca^2+^ influx was the result of activation of cationic mechanosensitive channels (MSCs) such as Piezo^11^,^12^, but inhibition of those channels with the specific inhibitor GsMTx4^13^ was not effective (Fig. 1f).

Based upon earlier reports of the mechanosensitivity of NMDA channels^6^, we tested the effect of MK-801, a specific pore blocker^14^. Pre-treatment of cells with MK-801 (50 μM, 8 min) almost completely inhibited the Ca^2+^ transients in ~75% of the cells (Fig. 1a/MK-801 panel) and reduced the peak response by ~45% in other cells (Fig. 1a/MK-801 panel, yellow arrows). Some cells did not respond to shear stress. The Ca^2+^ rise in all cells could be completely suppressed by adding 30 μM ruthenium red to 10 μM MK-801 (Fig. 1a/MK-801+ RR panel, 1b). Other NMDAR pore blockers, memantine^15^ (50 μM, Fig. 1c, light blue traces) and ketamine^16^ (50 μM, Fig. 1c, green traces), also inhibited the Ca^2+^ response. Low dose extracellular Mg^2+^ (2 mM) did not significantly affect the Ca^2+^ influx, but it is effective at high concentration (10 mM) (Fig. 1d). As a control for normal NMDAR activation, we gently perfused (<0.7 dyn/cm) cells with the agonists glutamate and glycine. This produced oscillatory Ca^2+^ responses (Fig. 1e, green traces) presumably arising from influx and calcium-induced-calcium-release (CICR)^17^. The glutamate activated Ca^2+^ increase was *completely suppressed* by MK-801 (Fig. 1e, blue traces, *P < 0.05 in Fig. 1f), whereas the shear induced response was only partially inhibited (Fig. 1f). Similarly, the inhibitory effect of memantine and ketamine was reduced under shear stimuli as though the efficacy had been reduced (Fig. 1f). Figure 1g summarizes the fraction of responding astrocytes to various treatments. The results suggest that the residual Ca^2+^ response observed with MK-801 under shear was due to either a parallel mechanically activated pathway^13^,^18^ or possibly a stress-induced change in the affinity for MK-801. A similar reduction in affinity was observed for Mg^2+^ inhibition (Supplementary Materials, SM1) as reported for reconstituted channels^6^.

Rat astrocytes have endogenous cationic MSCs inhibited by GsMTx4^13^,^19^ but we didn’t see evidence of their effect on the response to shear stress. We tested nonspecific inhibitors of MSCs including ruthenium red (RR)^20^ and Gd^3+^ 10. RR reduced the peak Ca^2+^ response by ~30% (Fig. 1a/RR panel, 1f), while Gd^3+^ blocked the response completely^10^. Why didn’t shear stress activate GsMTx4 sensitive Ca^2+^ fluxes when those MSCs are known to exist in the astrocytes? The most likely explanation is that shear stress doesn’t not produce the large tension in the bilayer characteristic of patch recording^7^,^21^.

If NMDARs are the primary contributor to the Ca^2+^ transients induced by shear forces, then NMDA in the presence of glycine should elicit inward currents in the astrocytes. In whole cell patch recordings, NMDA and glycine (100 μM each)^22^ did generate robust excitatory currents (Fig. 2). These currents were sensitive to a GluN2B-selective inhibitor (ifenprodil, 10 μM) and the glutamate antagonist (AP5, 100 μM). They were also inhibited by MK-801 (50 μM) and the conopeptide (NMB-1, 10 μM)^23^,^24^,^25^ (Fig. 2), so we conclude that functional NMDARs were present in our astrocytes. The presence of the GluN1 subunits in the astrocytes was further confirmed by Western blots (Supplemental Materials, SM2).

To test whether endogenous glutamate release might play a role in the mechanical activation of NMDARs^26^, we tested the response to shear stimuli in the presence of the binding site antagonists AP5 (50 to 200 μM) and ifenprodil (10 μM). In control experiments, they inhibited the response to glutamate and glycine (Fig. 3b,c), consistent with our patch clamp results, but they had no effect on the shear-induced Ca^2+^ increase (Fig. 3a,c). Thus, glutamate binding is not involved in the response to shear stress, but the pore blocker MK-801 did inhibit the response (Fig. 3a, blue). To see whether shear might cause glutamate secretion, we measured glutamate levels in the perfusate. Using an enzymatic fluorescence assay with NADH^27^, shear stimuli (23 dyn/cm^2^) did not produce a significant (>1 μM) increase of fluorescence (Supplemental Materials, SM3), while controls with glutamate added to the perfusate showed reliable responses at ~1 μM (Supplemental Materials, SM3)^27^. We conclude that glutamate binding is not involved in the NMDAR response to shear stress.

To try and associate the shear response with subunit specificity, we expressed GluN1,2A and GluN1,2B subunits in Chinese hamster ovary (CHO) cells. Wild type CHO cells responded to shear stress (23 dyn/cm^2^ for 10 ms) with a Ca^2+^ elevation but the response had a pronounced latency of ~6 s (Fig. 4a, gray). This response was completely blocked by 5 μM GsMTx4 (Fig. 4a blue) implying that CHO cells have functional Piezo type channels^18^.

We tested whether GluN1,2A or GluN1,2B expressing cells produced a shear sensitive Ca^2+^ flux with the endogenous MSCs inhibited by GsMTx4, and the Ca^2+^ response persisted in both cell types (Fig. 4a, red and orange). The Ca^2+^ response in cells expressing GluN1,2A was much higher than that for GluN1,2B, but both were inhibited by MK-801 (Fig. 4b,c). The peak Ca^2+^ amplitudes with shear stress were similar to those obtained with agonists without shear stress (Fig. 4b,c, green traces). The responses were also inhibited by MK-801 (Fig. 4b, blue). Note that the NMDAR responses differed from the endogenous MSC responses in that there was no significant latency between the stimulus and the initial onset of Ca^2+^ rise.

As expected from the astrocyte data, AP5 in CHO cells did not inhibit the response to shear stimulation of GluN1,2A (Fig. 4b, yellow), but both ifenprotil and AP5 inhibited the shear stress activated response of GluN1,2B (Fig. 4c, purple and yellow). This suggests that GluN1,2B is not responsible for the observed mechanosensitivity in rat astrocytes. The peak Ca^2+^ level results are summarized in Fig. 4d–f.

To examine the mechanical responses of NMDARs at higher resolution than in the shear stress experiments, we patch-clamped HEK cells transfected with GluN1,2A. Using outside-out patches we first tested NMDARs by perfusion with NMDA and glycine (Fig. 5b). We then applied positive pressure pulses and observed repeated channel openings that were in phase with the stimulus (Fig. 5). MK-801 mildly reduced the amplitude of these currents (Fig. 5a,c). Some patches showed multi-channel currents (n = 4) while others showed unitary currents (n = 3). The multi-channel currents were reduced to ~57 ± 14% (Mean ± s.e.m., n = 4, *P* < 0.1, Fig. 5a,c) with 50–100 μM MK-801 and this reduction was statistically significant. In the latter case, we measured the open probability (Po) of channels in the presence of MK-801 (50–100 μM) it was reduced to ~30 ± 11% (Mean ± s.e.m, n = 3, *P* < 0.05). Compared to the ~70% reduction of the Ca^2+^ response with the same dose of MK-801 seen with shear stress, the difference may be due to the much higher membrane tension in patches than in the shear stress experiments and distension of the pore with bilayer tension seems to alter the binding of both MK-801 and Mg^2+^ in the pore. The inhibition remained dependent on the concentration of MK-801. As a control for a generic mechanical response to MK-801, we expressed hPiezo1 (which we suspected to be a background current), and applied NMDA or MK-801 but we did not observe any inhibition.

## Discussion

NMDARs are mechanically sensitive in the absence of agonists. We visualized this behavior by using fluid shear stress as a uniform, precise and gentle mechanical stimulus. The results supported earlier data that showed channels activated by mechanical stimuli including anisotonic solutions, or stretching patches and the substrate^4^,^28^. Most of those studies presumed that the stretch-sensitive component of the kinetics represented the properties of liganded channels^4^,^5^,^28^. Now we have learned that unliganded channels are mechanically responsive. This fits with observations on reconstituted NMDARs^6^.

While the gating properties are obviously mechanically sensitive, there is a suggestion that pore dimensions may also be modified as suggested by data from reconstituted channels^6^ and mechanically injured neurons^5^. The putative pore effects are visualized as reduction in the ability of Mg^2+^ to inhibit permeation of Ca^2+^ and monovalent cations^29^. In our experiments 2 mM extracellular Mg^2+^ had a minimal effect on shear induced cell Ca^2+^ influx, although higher concentrations did significantly inhibit. This dampening of the inhibitory effect of Mg^2+^ may be the result of membrane tension expanding the pore diameter, as has been suggested for reconstituted channels. As we stated, our stress-induced responses were only *partially* inhibited by saturating doses (50 μM) of MK-801, but were completely inhibited with a mixture of MK-801 and Ruthenium Red. Since MK-801 is an efficient pore blocker, this reduction in MK-801 sensitivity with shear stress may be due to the postulated deformation of the pore and may explain the rapid dissociation rate we observed.

The magnitude of fluid shear we used did not significantly deform the cells (see Supplemental Materials, SM4) raising the obvious question of how does fluid flow modulate NMDARs? Fluid shear by itself is not likely to directly affect the bilayer since most of the velocity gradient is absorbed by the glycocalyx leaving little fluid velocity at the bilayer^30^. However, fluid drag applied to the ECM will pull on the cytoskeleton increasing its stress^31^,^32^, and bilayer tension is known to be coupled to cytoskeletal tension^31^,^32^. While our fluid velocity was precisely controlled, the body stress it produced in the cells depends upon cell geometry and the distribution of stress within the cytoskeleton; that is known to be heterogeneous^33^,^34^ and this heterogeneity likely accounts for the variability between cells.

An inherent difficulty in studying biomechanics is that there is no way to apply uniform local stresses^35^, but we can make a rough estimate of cortical tension. In mammalian cells, Heureaux *et al*.^36^ used a precalibrated probe of bilayer tension, the bacterial mechanical channel MscL, and found that the fluid shear stress they used would not activate MscL. Their predicted peak stress was twenty fold greater than what we used. Since MscL gates with a tension of ~5 mN/m^37^, the flow induced increase in bilayer tension under our conditions is likely much less than 5 mN/m. If we linearly extrapolate Heureaux *et al*.’s data to our conditions, we predict we had a peak bilayer tension <0.3 mN/m. Haidekker *et al*.^38^ did an independent measurement of bilayer tension under fluid shear using fluorescent probes to measure membrane fluidity. Changes in fluidity match changes in tension^39^. In endothelial cells they found that a shear stress of 26 dyn/cm^2^ increased the bilayer fluidity (or equivalently increased its tension) by 22%. Using Haidekker’s probes, we found that our fluid shear increased the bilayer tension by only ~10% over that at rest, and since the resting bilayer tension is small^8^, the fluid stress that we used appears to be too small to activate traditional MSCs like Piezo1^40^.

Our precision shear pulses showed that many astrocytes responded with a Ca^2+^ rise that began without a measurable latency. The overall Ca^2+^ responses included both influx and release from ER stores. The initial Ca^2+^ rise and the subsequent transient was eliminated in Ca^2+^-free media, but it was present after depleting Ca^2+^ stores with thapsigargin^10^. Why should it take seconds for the Ca^2+^ response to peak following a 10 ms pulse? There seem to be two possibilities: 1) the initial influx of Ca^2+^ raises local Ca^2+^ concentration activating a second messenger cascade such as CICR, or 2) the induced body stress caused a break of some cytoskeletal links and that led to plastic deformation of the cytoskeleton that was eventually coupled to bilayer tension. We do not yet know whether NMDARs *in vivo* are activated by bilayer tension or by direct coupling to the cytoskeleton. Martinac’s experiments on reconstituted NMDARs^6^ shows that bilayer tension alone was adequate to open the reconstituted channels. However, the literature shows NMDARs linked to α-actinin^41^, spectrin, and other actin binding proteins^42^ and that could also couple force to the channel^37^.

**Figure 4:**
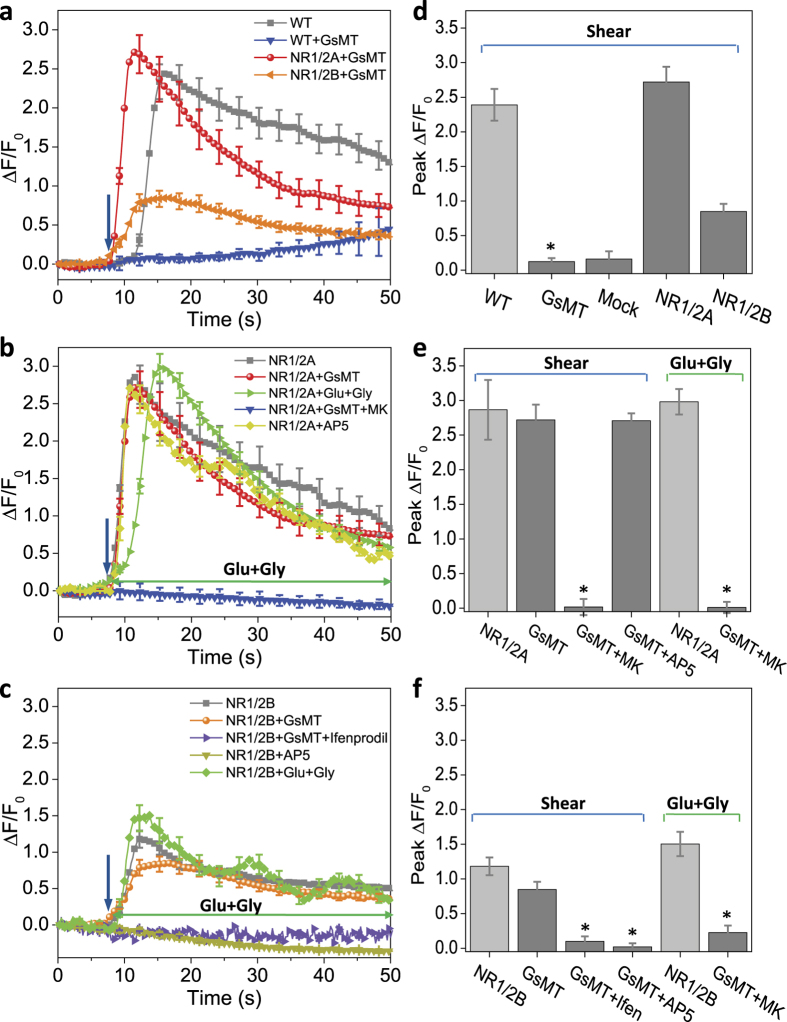
Ca^2+^ response to fluid shear stimuli in GluN1,2A and 2B expressing CHO cells. (**a**) The Ca^2+^ response to a shear pulse (23 dyn/cm^2^, 10 ms) in control cells (wild type, gray), with MSC inhibitor GsMTx4 (blue), and GluN1,2A (red) and GluN1,2B (orange) expressing cells in the present of GxMTx4, showing recombinant NMDA channels can be activated by shear stress without agonists. (**b**) Ca^2+^ response to shear pulse and glutamate-glycine (green) in GluN1,2A expressing cells, showing that glutamate antagonists do not inhibit shear responses. Results for the GluN1,2A expressing cells (red) are replotted for comparison. (**c**) Ca^2+^ response to shear pulse and glutamate-glycine (green) in GluN1,2B expressing cells, showing glutamate biding sites play a role on N2B subunit. Each curve is an average over 200 cells from >4 experiments under each condition. Error bars indicate s.e.m. (**d–f**) Peak Ca^2+^ response in each conditions corresponding to (**a–c**), respectively. Cells treated with various drugs were compared with control cells with corresponding stimuli (*p < 0.05). Error bars indicate s.e.m.

Based upon the CHO expression studies, GluN1,2A seems to be the subunit that accounts for mechanosensitivity (Fig. 4). These subunits are expressed in cortical astrocytes *in vivo* and in acute brain slices^43^,^44^,^45^,^46^. Astrocytes themselves are heterogeneous and different subunits are expressed in different parts of the brain^47^. The Ca^2+^ responses of our primary adult rat astrocytes to both agonists and shear stress were similar to responses from acutely isolated cortical astrocytes from day old rats.

Mechanical signals play roles in the CNS including in brain development^48^ and possibly perfusion of brain tissue by the glymphatic system^49^. Our results may have implications for the treatment of TBI, since therapies may require a focus on improving the distribution of cytoskeletal stress. In adult mice under anesthesia, glymphatic flow increases^49^ but anesthesia reportedly also decreases astrocyte Ca^2+^ 50, in apparent contradiction to our hypothesis. However, the anesthesia used by Thrane *et al*.^50^ contained ketamine, which is an inhibitor of NMDARs. While we have emphasized the potential role of NMDARs in the CNS, NMDARs are also present outside the CNS as in the postsynaptic folds of skeletal muscle^51^. In a mechanically active tissue like muscle the mechanical sensitivity may dominate the glutamate sensitivity.

## Materials and Methods

### Flow chamber and shear stimuli

The microfluidic flow chamber is a parallel plate flow system with a 25 mm diameter cover glass substrate and a 1 mm thick glass slide separated by Polydimethylsiloxane (PDMS) walls. The chamber dimensions are 1000 μm wide, 15 mm long and 100 μm high. For application of various drugs, we used a chip consisting of three inlet channels that merged into the main flow. The device was fabricated using soft lithography following a fabrication process described previously^52^. The chamber substrate was coated with human fibronectin (BD Bioscience) and rinsed an hour before seeding the cells. A fast piezo-controlled pressure servo (ALA Scientific Instruments, NY) was used to generate pressure pulses of known waveform with a time resolution of ~1 ms^9^. To mimic blast waves in the fluids, we used a waveform resembling the classic Friedlander curve^53^. The fluid shear stress in the chamber was previously calibrated^10^.

### Cell culture and transfection

Primary adult astrocytes were obtained using gelatin-sponge implants from adult Sprague Dawley rat brains provided by Dr. Thomas Langan (SUNY Buffalo). Cells were maintained in DMEM, 10% fetal bovine serum and 1% Penicillin/Streptomycin. To permit phenotypic maturation of astrocytes, they were used in experiments between passages 3 and 10. For the Ca^2+^ assays, cells were transferred to the microfluidic chambers when the culture flasks reached 95% confluence, and they were cultured in the chamber for 3–5 days before the experiments. Media in the chamber was changed every 24 hrs to sustain the cell growth. For electrophysiology, cells were plated at high densities (80–95%) and maintained in 35 mm dishes for 1–3 days. Prior to electrophysiological recordings, cells were suspended and replated at low density (<5%) onto separate dishes with 2 mL fresh DMEM supplemented with 2 mM MgCl_2_. Cells were allowed to adhere to plates for 30–60 minutes at 37 °C. Prior to electrophysiological recordings, the growth medium was replaced with PBS.

Chinese Hamster Ovary cells (CHO-K1, ATCC) were maintained in F-12 K medium containing 10% fetal bovine serum and 1% Penicillin/Streptomycin. CHO cells were co-transfected with plasmids of GluN1 (0.8 μg) and GluN2A (0.8 μg) or GluN1 (0.8 μg) and GluN2B (0.8 μg) using Effectene Transfection Reagent (QIAGEN, Valencia, CA). CHO was chosen as model cell for expressing NMDA receptors because the properties of transfected receptors were similar to endogenous channels in neurons^54^. Cells were kept in medium containing transfection reagent and plasmids for ~24 hours, then switched to normal medium for another 24 hr before the experiments. The transfection efficiency was ~40%.

### Electrophysiology

Whole-cell currents were recorded using borosilicate glass pipettes (2–5 MΩ) filled with (in mM) 135 CsCl, 33 CsOH, 2 MgCl_2_, 11 EGTA, 1 CaCl_2_ and 10 HEPES (pH 7.4). Cells were first perfused with a wash solution containing (in mM): 150 NaCl, 2.5 KCl, 0.01 EDTA, 0.5 CaCl_2_, 0.1 glycine, and 10 HEPBS (pH 8.0) followed by solutions supplemented with NMDA (100 μM, duration 5 s) and with NMDA and drug (5 s), as indicated. Cells were held at −70 mV; currents were filtered at 2 kHz (Axopatch 200B), sampled (Digidata 1440 A) and stored as digital files (pClamp 10.2) for off-line analysis.

Outside-out patches were made from HEK293 cells transfected with GluN1A/2A or hPiezo1 cDNA. Only those patches which responded to NMDA were considered for analysis. NMDARs were mechanically-activated by a pulse train of 500 ms positive pressure steps separated by rest periods of 1500 ms. The bath and pipette solutions were the same as those used for whole-cell recording. The outside-out patches were tested at +50 mV membrane potential because with positive patch pressure depolarized potentials yielded more stable patches. The drugs, NMDA (200 μM) or MK-801 (50–500 μM) in bath solution, were perfused directly onto the outside-out patches. Analysis was performed offline using QuB software (www.qub.buffalo.edu). The mean currents were obtained by time integration of the current (a measure of total charge transferred).

### Cytosolic Ca^2+^ and extracellular glutamate measurements

Prior to the experiments, cells were gently washed with saline followed by loading with the Ca^2+^ sensitive dye, Fluo-4 AM (5 μM, Invitrogen), and kept in an incubator (37 °C with 95% air and 5% CO_2_) for 30 min. The chambers were then gently washed and returned to the incubator for ~8 min to allow cleavage of the dye molecules. Normal saline was used as the shearing fluid. Fluorescence images were acquired using an inverted microscope (Axiovert 200 M, Zeiss) equipped with a CCD camera (AxioCam MRm, Zeiss) and a 10x objective lens. A filter set (Ex: 470/40 nm; Em: 525/50 nm) was used for Ca^2+^ imaging. Time-lapse images were obtained using Zeiss software (AxioVision, Ziess). The concentration of glutamate in solution was measured using the established assay based on the reduction of NAD^+^ to NADH in the presence of glutamate with a filter set (Ex: 365; Em: 445/50)^27^.

### Protein extraction and Western blot

Cells were washed twice in cold PBS and lysed into 100 mM Tris pH 7.4, 5 mM EDTA, 150 mM NaCl, 1% SDS, 1 mm Na_3_VO_4_, 10 mm NaF, with protease inhibitors. Protein quantities were determined using microBCA assay (Thermo Scientific). We loaded 100 μg protein lysate from primary astrocytes and 20 μg from HEK293 cells. Blotted membranes were blocked for 1 hr with 5% BSA and probed overnight at 4 °C with Millipore Rbt anti-NR1 1/1000 (MAB1586) in 1% BSA TBST 0.05%. Membranes were rinsed in TBST 0.05% incubated for 1 h with a secondary antibody (Santa Cruz Gt anti-mouse HRP (sc-2062)). Blots were developed using ECL plus (GE Healthcare). Western blots were performed at least twice. For a loading control, membranes were stained with Amido Black (Sigma).

### Immunofluorescence and immunohistochemistry

For GFAP immunostaining, rat primary cells were fixed with 4% ice-cold paraformaldehyde for 15 min and washed three times with PBS. Cells were then permeabilized with 0.1% Triton in PBS for 10 min at 37 °C and blocked overnight in 5% NGS at 4 °C. Cells were incubated for 1 hr at 37 °C with Santa Cruz GFAP 1/500 (sc-6170) and for 1 h at 37 °C with Donkey anti rabbit TRITC 1/500 (711-025-152). Images were acquired with a fluorescent microscope, Leica DM6000B.

### Solutions and reagents

Normal saline containing 1 mM CaCl_2_ was the control solution. Ruthenium Red, DL-2-amino-5-phosphonopentanoic acid, (+)-MK-801 hydrogen maleate, and Memantine hydrochloride (all from Sigma, St. Louis, MO) were diluted, respectively, to 30 μM, 100 μM and 50 μM. Ketamine hydrochloride (Zoetis) was diluted to 50 μM. Glutamate and Glycine (Sigma, St. Louis, MO) were diluted to 1 mM in saline. The enantiomeric forms of GsMTx4 were made and purified according to previously published protocols^55^. L-Glutamic Dehydrogenase from Bovine Liver (G2626) and β-Nicotinamide adenine dinucleotide hydrate (N7004) (Sigma, St. Louis, MO) were diluted respectively to 56 U/ml and 1 mM in saline and pre-incubated with the cells before the glutamate assay^25^.

### Data Analysis

Relative Ca^2+^ intensity was calculated using 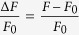, where *F* and *F*_*0*_ are the mean fluorescence intensities of selected cells at time t and t = 0, respectively. The background was subtracted before the calculation. The mean calcium changes were an average over N selected cells from each image and from multiple experiments. A new cell culture was used for each experiment. The statistical analysis used the standard error of the mean (s.e.m.). Statistics of peak Ca^2+^ intensity under different drug treatments were compared with control cells subjected to corresponding stimuli, and analyzed with a paired Student’s *t*-test where *p* < 0.05 was considered significant. For electrophysiology, steady-state current amplitudes in the absence (I_ss_) or presence of drug (I_Drug_) were determined by fitting a single exponential function to the declining portion of the current trace. The extent of response was determined according to the equation: % current response = (I_Drug_/I_ss_) × 100. Differences between I_Drug_ and I_ss_ were considered significant for *p* < 0.05 according to a paired Student’s *t*-test.

## Additional Information

**How to cite this article**: Maneshi, M. M. *et al*. Mechanical stress activates NMDA receptors in the absence of agonists. *Sci. Rep.* **7**, 39610; doi: 10.1038/srep39610 (2017).

**Publisher's note:** Springer Nature remains neutral with regard to jurisdictional claims in published maps and institutional affiliations.

## Supplementary Material

Supplementary Information

## Figures and Tables

**Figure 1:**
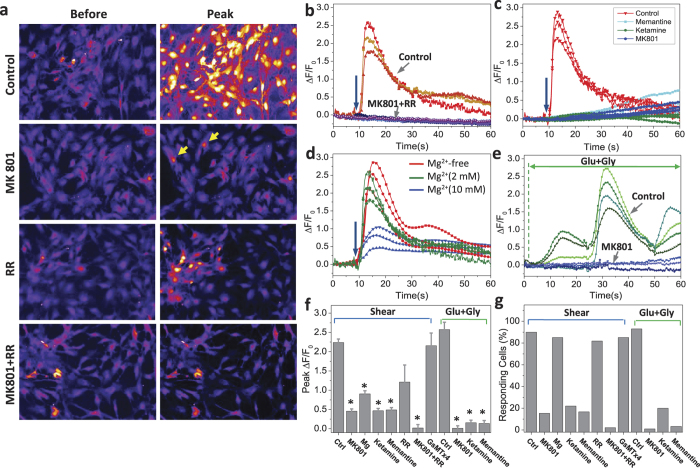
Sources of shear-activated Ca^2+^ increase in astrocytes using pharmacology. (**a**) Fluorescence images of Fluo-4 loaded astrocytes before the stimulus pulse (23 dyn/cm^2^, 10 ms) and 6 s later at the peak of the response under control conditions and with treatments of various inhibitors. Yellow arrows indicate the responding cells. (**b**) Time dependent changes in Ca^2+^ in controls and with a mixture of MK-801 (10 μM) and Ruthenium red (30 μM). Each trace was measured from selected single cell. The shear stimulus was applied at the time indicated by the arrow. The data show that the mixture completely blocked the Ca^2+^ response. (**c**) The Ca^2+^ response in cells treated with MK-801 (dark blue traces, from cells in the image of panel a, section MK-801), memantine (light blue traces), and ketamine (green traces), compared with control cells (red traces). (**d**) The Ca^2+^ response in cells treated with 0, 2 and 10 mM Mg^2+^, showing NMDAR sensitivity to low dose Mg^2+^ (2 mM) was reduced by shear stress. (**e**) Ca^2+^ response to agonists, glutamate (1 mM) and glycine (1 mM) (green curves). MK-801 (50 μM) blocked the agonist activated Ca^2+^ response (blue curves). (**f,g**) Summary of peak Ca^2+^ responses **(f)** and the number of responding cells (**g**) in cultures treated with different drugs. The means are from N = 200 cells from >4 experiments under each condition. Cells treated with various drugs were compared with control cells with the same stimuli (*p < 0.05). Error bars indicate s.e.m.

**Figure 2:**
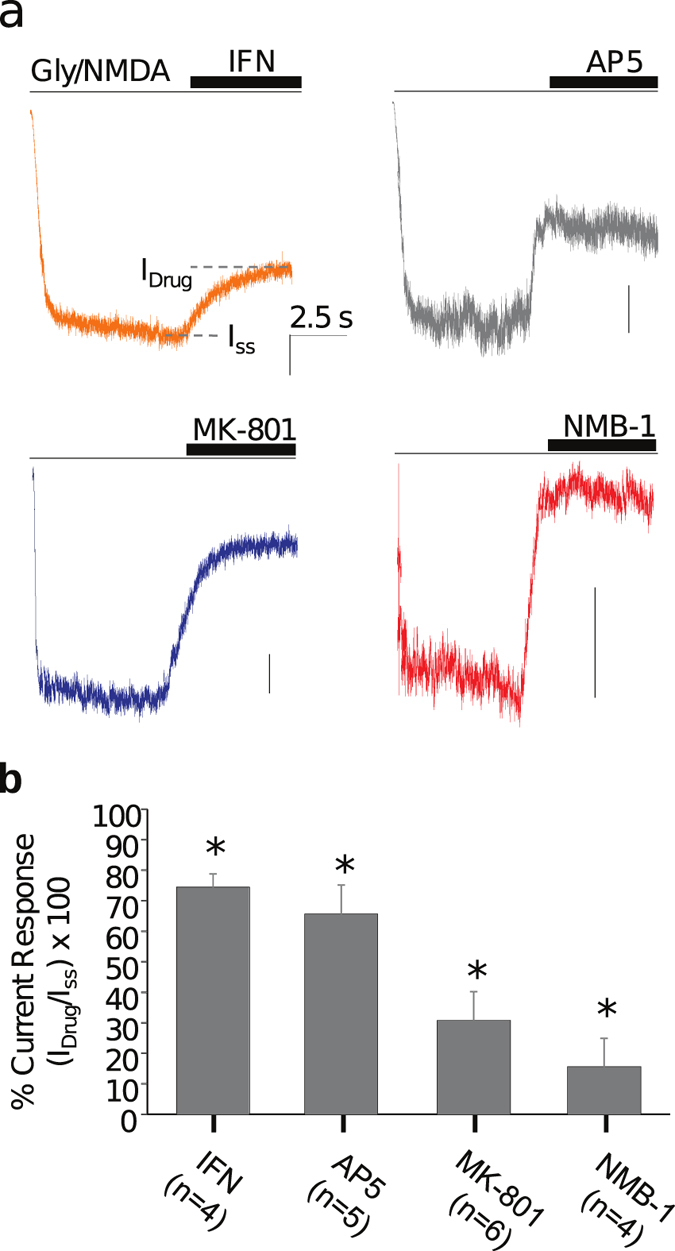
Pharmacological inhibition of NMDA-evoked currents in cultured astrocytes. (**a**) Representative whole-cell currents evoked by co-application of NMDA (100 μM) and glycine (100 μM); drugs were applied for 5 s to reach steady-state (I_ss_). Vertical scale bars represent 100 pA. (**b**) Summary of current blockage by IFN (10 μm, N = 4), AP5 (100 μM, N = 5), MK-801 (50 μM, N = 6), and NMB-1 (10 μM, N = 4); *Indicates significant differences relative to currents in the absence of drugs (*p* < 0.05).

**Figure 3:**
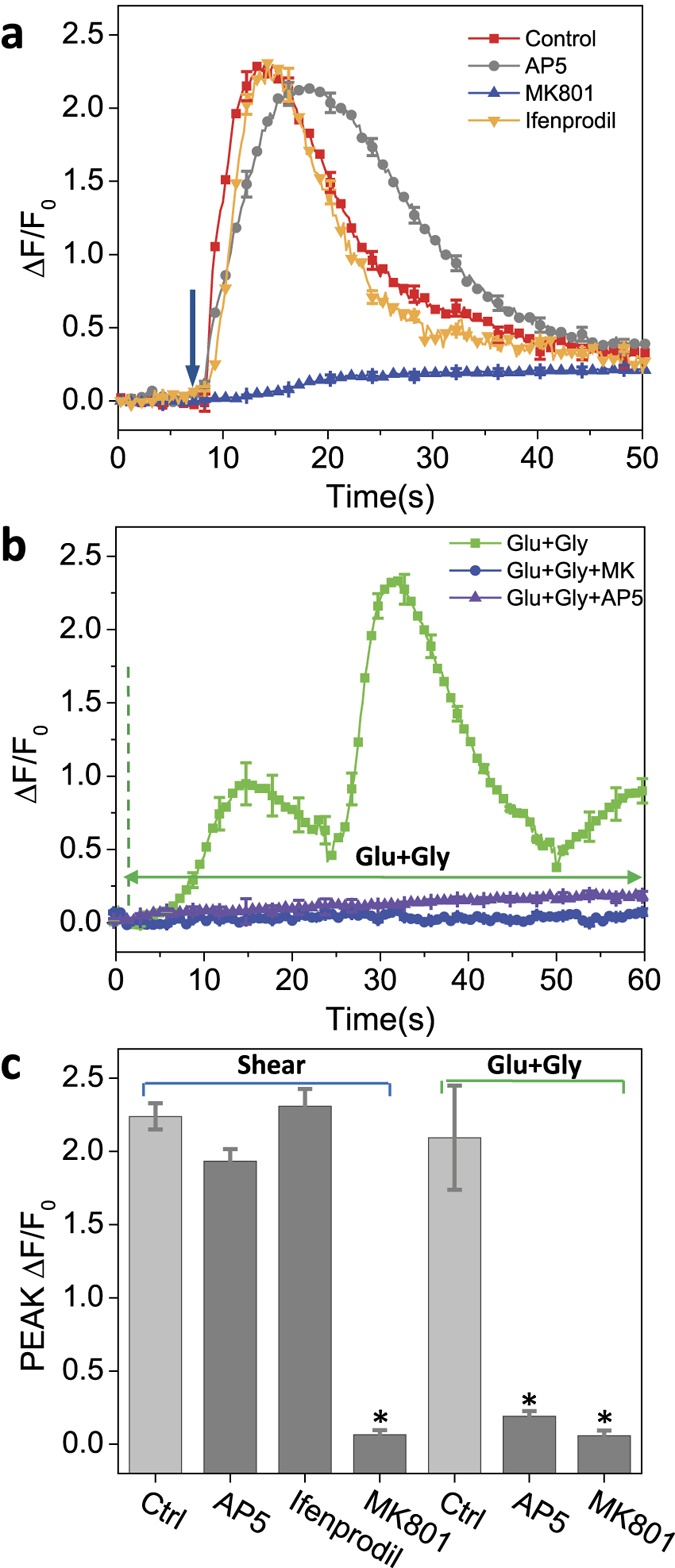
Role of free glutamate and glutamate binding sites on shear induced Ca^2+^ response in astrocytes. (**a**) Ca^2+^ response to shear pulse stimuli (23 dyn/cm^2^, 10 ms) in control (red), AP5 (50 μM, Gray), IFN (10 μm, orange), and MK-801 (50 μM, Blue) showing that blocking glutamate binding sites did not affect the Ca^2+^ response to shear. **(b)** Ca^2+^ response to agonists (1 mM glutamate and 1 mM glycine) in control (green), AP5 (200 μM), and MK-801 (50 μM). Each curve was an average of 200 cells from 4 experiments (*p < 0.05). **(c)** Peak Ca^2+^ intensity with and without glutamate inhibitors. Error bars indicate s.e.m.

**Figure 5:**
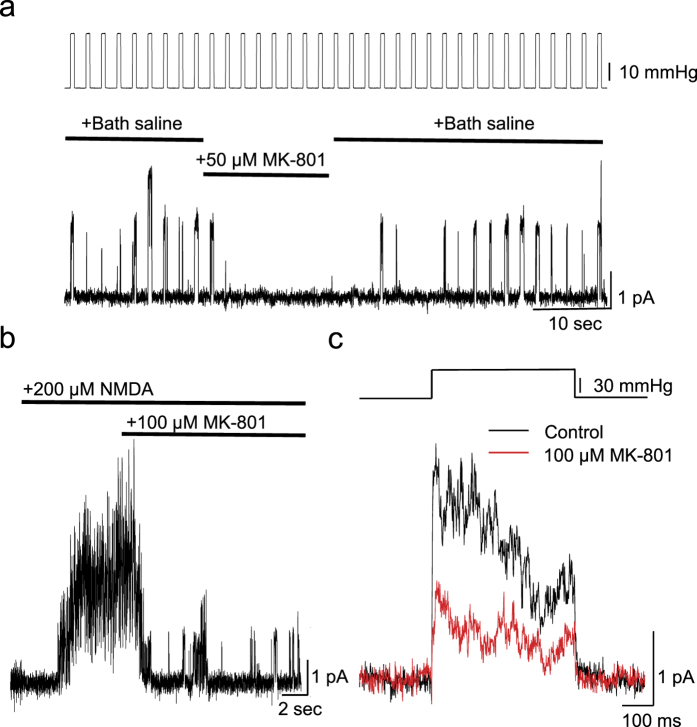
Mechanically-activated NMDAR current in outside-out patches. Patches that showed channel activation in response to NMDA were used for further analysis. (**a**) Pulse train showing mechanically-activated unitary current from NMDARs and inhibited by 50 μM MK-801. (**b**) Block of NMDA (200 μM) activated current by MK-801 (100 μM) with patch at rest. (**c**) Representative traces showing MK-801 inhibition of a pressure pulse response (~60 mmHg) (n = 4, independent patches form different culture dishes).
