# Supplementary material for: Mechanical stress activates NMDA receptors in the absence of agonists
Source: Sci Rep. 2017 Jan 3;7:39610. doi: 10.1038/srep39610 (PMC5206744; doi:10.1038/srep39610)

Mechanical stress activates NMDA receptors in the absence of agonists

Mohammad Mehdi Maneshi,[1,2] Bruce Maki,[3,‡] Radhakrishnan Gnanasambandam,[1,‡]

[illegible],[3] Gabriela K. Popescu,[3] Frederick Sachs,[1] and Susan Z. Hua[1,2,]*

[1]Department of Physiology and Biophysics, [2]Department of Mechanical and Aerospace Engineering,

[3]Department of Biochemistry, SUNY-Buffalo, Buffalo, New York 14260, USA

Supplemental Materials

SM 1. Dose dependence of Mg^{2+} inhibition for NMDA and shear stimuli. Cells were pre-treated with Mg^{2+} at given concentration for 5 min., followed by application of shear stimulus (23 dyn/cm^2, 10 ms) or gentle perfusion of NMDA and Glycine (100µM each). The peak Ca^{2+} changes were measured from n=30 cells from two cultures for each concentration. This data shows that the inhibitory effect of Mg^{2+} is reduced under mechanical forces. The data was fit to Boltzmann equation (R^2 ~ *0.98*, dashed curves).

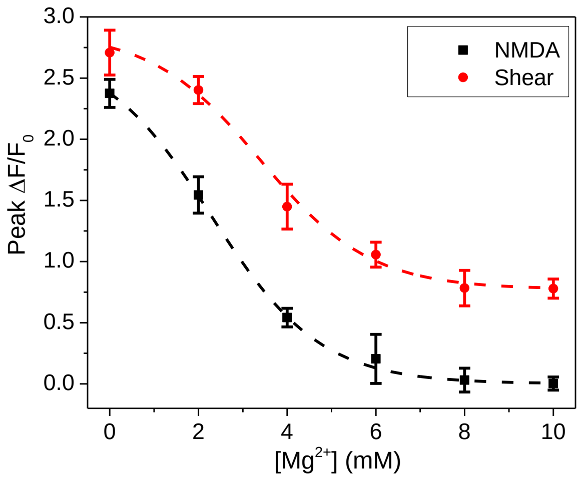

SM 2. Expression of NMDA subunits in primary astrocytes. (a) Immunofluorescence staining for glial fibrillary acidic protein (GFAP) of purified primary rat astrocytes (30 DIV). Left: Merged image of GFAP (green) and DAPI staining; Right: DAPI staining (blue); Middle: Secondary control Goat anti rabbit 488 (magnification: 40×). **(b)** Detection of NMDAR NR1 subunit in astrocytes with immunoprecipitation. Primary astrocytes' total protein lysate (100ug) were separated by SDS/PAGE and probed for pan-NR1 (120 kDa). Control and recombinant protein NR1-1a expressing HEK293 cells were used as positive and negative controls (20 μg). Amido black was used as loading control.

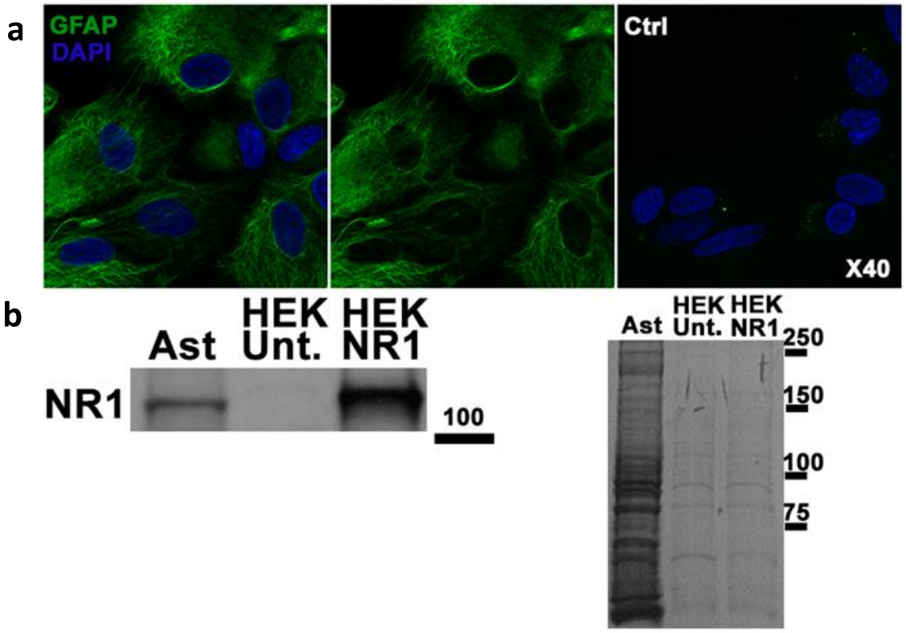

SM 3. Measurements of glutamate level in solution. (a) Bright field of a single astrocyte and NADH fluorescence images before and after shear pulse stimulation, showing that the release of glutamate from cells is below the noise level (~1 μM). **(b)** Time course of NADH fluorescence intensity. A shear pulse (23 dyn/cm^2, 10 ms) is applied at the time indicated by arrow. **(c)** Calibration of NADH fluorescence against various glutamate levels in solution, showing the assay can detect 1 μM change of glutamate in solution.

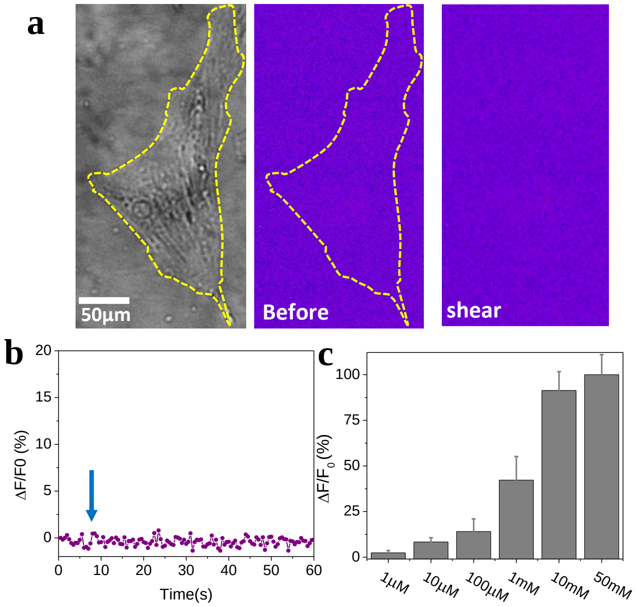

SM 4. Astrocyte deformation under fluid shear stress. (a, b) Fluorescent images (CFP fluorescence channel) of an astrocyte labeled with actinin-FRET probes before and under a fluid shear pulse (23 dyn/cm^2, 400 ms). The shear stress pulse was applied at t=0 ms, the arrow indicates flow direction. **(c)** Overlap of two intensity masks generated from (a, red) and (b, green) using ImageJ, showing the deformation is non-uniform within a single cell. **(d)** The cell deformation was estimated using $\varepsilon=\Delta D/D_0$, where D and D_0 are the mean cell diameters at t = 0 and 380 ms, respectively, showing maximal strain is ~ 4%.

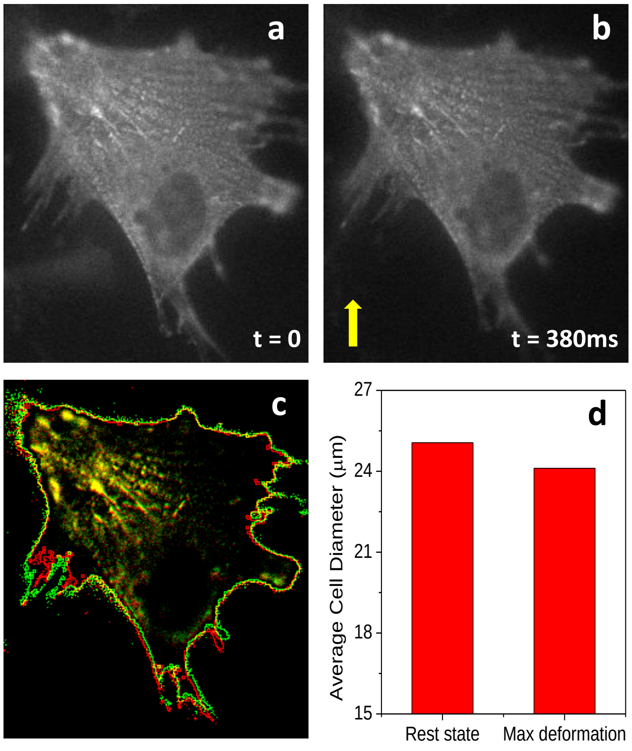

Supplement: Supplementary Information [file srep39610-s1.pdf]
